# Magnetic Resonance Elastography reveals effects of anti-angiogenic glioblastoma treatment on tumor stiffness and captures progression in an orthotopic mouse model

**DOI:** 10.1186/s40644-020-00314-1

**Published:** 2020-05-12

**Authors:** Katharina Schregel, Michal O. Nowicki, Miklos Palotai, Navid Nazari, Rachel Zane, Ralph Sinkus, Sean E. Lawler, Samuel Patz

**Affiliations:** 1 Department of Radiology, Brigham and Women’s Hospital, Boston, MA USA; 2 Harvard Medical School, Boston, MA USA; 3 Department of Neuroradiology, Heidelberg University Hospital, Heidelberg, Germany; 4 Institute of Neuroradiology, University Medical Center Goettingen, Goettingen, Germany; 5 Harvey Cushing Neurooncology Laboratories, Department of Neurosurgery, Brigham and Women’s Hospital, Boston, MA USA; 6 Department of Biomedical Engineering, Boston University, Boston, MA USA; 7 School of Biomedical Engineering and Imaging Sciences, King’s College London, London, UK; 8 INSERM U1148, Laboratory for Vascular Translational Science, University Paris Diderot, University Paris 13, Paris, France

**Keywords:** Glioblastoma, Anti-angiogenic treatment, Magnetic resonance imaging, Magnetic resonance elastography, Tumor stiffness, Anti-VEGF antibody

## Abstract

**Background:**

Anti-angiogenic treatment of glioblastoma (GBM) complicates radiologic monitoring. We evaluated magnetic resonance elastography (MRE) as an imaging tool for monitoring the efficacy of anti-VEGF treatment of GBM.

**Methods:**

Longitudinal studies were performed in an orthotopic GBM xenograft mouse model. Animals treated with B20 anti-VEGF antibody were compared to untreated controls regarding survival (*n* = 13), classical MRI-contrasts and biomechanics as quantified via MRE (*n* = 15). Imaging was performed on a 7 T small animal horizontal bore MRI scanner. MRI and MRE parameters were compared to histopathology.

**Results:**

Anti-VEGF-treated animals survived longer than untreated controls (*p* = 0.0011) with progressively increased tumor volume in controls (*p* = 0.0001). MRE parameters viscoelasticity |G*| and phase angle Y significantly decreased in controls (*p* = 0.02 for |G*| and *p* = 0.0071 for Y). This indicates that untreated tumors became softer and more elastic than viscous with progression. Tumor volume in treated animals increased more slowly than in controls, indicating efficacy of the therapy, reaching significance only at the last time point (*p* = 0.02). Viscoelasticity and phase angle Y tended to decrease throughout therapy, similar as for control animals. However, in treated animals, the decrease in phase angle Y was significantly attenuated and reached statistical significance at the last time point (*p* = 0.04). Histopathologically, control tumors were larger and more heterogeneous than treated tumors. Vasculature was normalized in treated tumors compared with controls, which showed abnormal vasculature and necrosis. In treated tumors, a higher amount of myelin was observed within the tumor area (*p* = 0.03), likely due to increased tumor invasion. Stiffness of the contralateral hemisphere was influenced by tumor mass effect and edema.

**Conclusions:**

Anti-angiogenic GBM treatment prolonged animal survival, slowed tumor growth and softening, but did not prevent progression. MRE detected treatment effects on tumor stiffness; the decrease of viscoelasticity and phase angle in GBM was attenuated in treated animals, which might be explained by normalized vasculature and greater myelin preservation within treated tumors. Thus, further investigation of MRE is warranted to understand the potential for MRE in monitoring treatment in GBM patients by complementing existing MRI techniques.

## Background

Glioblastoma (GBM) is the most common malignant primary brain tumor [1]. It has a very poor prognosis with less than 10% of patients surviving longer than 5 years when treated with the standard of care regimen, which comprises surgical resection followed by radiotherapy and concomitant alkylating chemotherapy with temozolomide [2]. Important hallmarks of GBM are highly invasive growth, regional heterogeneity, necrosis and microvascular proliferation [3]. Magnetic resonance imaging (MRI) is an important tool for diagnosis and monitoring of GBM. The current recommendations of the Response Assessment in Neuro-Oncology (RANO) working group include evaluation of both enhancing and non-enhancing tumor components on contrast-enhanced T1- and T2-weighted (T1w, T2w) or fluid-attenuated inversion recovery (FLAIR) MRI sequences [4]. However, the evaluation of non-enhancing tumor components remains controversial and is therefore not recommended as a radiographic endpoint in clinical GBM trials [5]. Thus, radiological response assessment is challenging and must be done carefully.

Magnetic resonance elastography (MRE) is an MR imaging technique that renders objective quantifiable measures of biomechanical tissue properties non-invasively and in vivo [6]. For MRE, mechanical vibrations are applied to the tissue of interest which induce mechanical shear waves. The propagation of these waves in space and time is measured using a motion encoding gradient and the biomechanical properties are calculated from the displacement field [7]. MRE of the brain is feasible and safe [8] and has been used to quantify the biomechanical properties of a variety of brain tumors [9]. Malignant brain tumors are softer than healthy brain parenchyma [10, 11] and the stiffness of gliomas inversely correlates with tumor grade [12], with GBM being softer than lower grade gliomas. MRE revealed that GBM has a high intratumoral heterogeneity with regard to its biomechanical composition [13]. Moreover, GBM subregions with different histopathological features could be identified based on their viscoelastic properties [14] and a correlation between MRE parameters and cellular and microvessel density could be observed in a preclinical study comparing three different brain tumor models [15]. The effect of therapeutic intervention on GBM stiffness has not been the subject of any clinical or preclinical investigation so far, except for one study evaluating the effects of radiotherapy on tumor stiffness in a murine model of GBM [16]. Hence, studies focusing on the effects of treatment on biomechanical tumor properties are needed to determine whether MRE could potentially complement radiological tumor monitoring in the future.

Effects of therapeutic agents can complicate the evaluation of GBM progression with MRI. For example, anti-angiogenic treatment of GBM that blocks vascular endothelial growth factor A (VEGF-A or VEGF), a critical proangiogenic mediator, causes a “normalization” of the vasculature [17] and leads to reduced cerebral edema and improved drug delivery to the tumor [18]. However, anti-VEGF treatment also leads to a phenomenon termed “pseudoresponse”, which describes a decrease in contrast enhancement on T1w MR images in the absence of a real antitumor effect of the treatment [19, 20].

Therefore, the objective of this preclinical pilot study was to investigate whether biomechanical GBM parameters are affected by anti-angiogenic treatment and whether MRE is capable of differentiating treated tumors from untreated controls. If MRE could help to better distinguish a true tumor response from pseudoresponse, a definite diagnosis could be established earlier. Currently, phenomena such as pseudoresponse are best diagnosed through follow-up scans [19], potentially delaying treatment decisions.

This preliminary study is intended to provide initial data to support further detailed studies on the ability of MRE to provide information on treatment response or tumor progression, both of which are sometimes indiscernible on standard MR images.

## Materials and methods

### Animal model

All experiments were performed in accordance with the local institutional animal care and use committee (IACUC) and the U. S. National Institutes of Health guidelines for the care and use of Laboratory animals. Results were reported according to the ARRIVE guidelines [21]. The glioblastoma stem cell line (G9pCDH) used for this study was provided by The Ohio State University Tissue Procurement services. The cell line was originally established from a patient tissue sample obtained under an IRB approved protocol at The Ohio State University Medical Center. The G9 line was stably transduced with pCDH lentiviral vector (cat# CD511A-1, System Biosciences, Palo Alto, CA, USA), to allow expression of copGFP, a highly stable and bright green fluorescent protein.

Tumor cells (50,000 per animal) were surgically implanted into the right central striatum of 28 female athymic nude mice (nu/nu, Envigo, South Easton, MA, USA) using the following coordinates: 2 mm right lateral and 0.5 mm frontal to the bregma at 3.5 mm depth [22].

### Study design

**Fig. 1:**
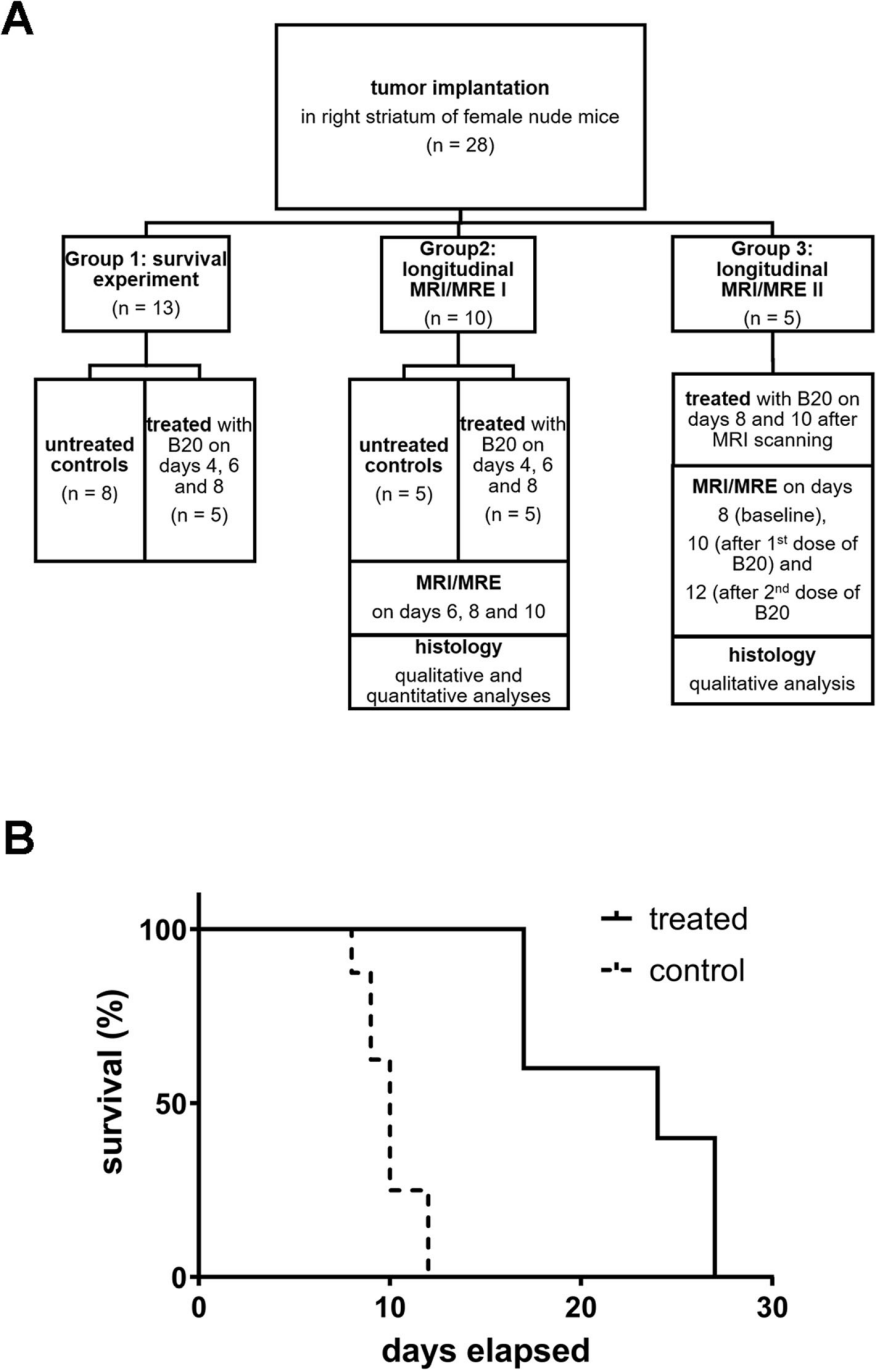
A flow chart depicts the different experiments and experimental groups (**a**) and provides an overview of the study design. An initial experiment compared the survival of animals treated with the B20 anti-VEGF-antibody and untreated controls. Survival analysis was performed according to the Kaplan-Meier product limit method and a survival curve is shown (**b**). Treated animals (black line) survived significantly longer than untreated controls (dotted line; median survival with and without treatment 24 and 10 days; *p* = 0.0011)

The animals were divided into three different experimental groups. A schematic overview of the experimental design for each group is provided in Fig. 1a. The first group determined the survival time of five animals that received intraperitoneal (i.p.) injections of 10 mg B20 anti-VEGF antibody 4, 6 and 8 days after tumor implantation and was compared to eight untreated animals. Then, experiments that included MRI and MRE were conducted in two additional groups (groups 2 and 3). The experiment in group 2 consisted of a longitudinal comparison between five untreated control animals and five animals that received i.p. injections of 10 mg B20 anti-VEGF antibody 4, 6 and 8 days after tumor implantation. MRI and MRE were performed 6, 8 and 10 days after implantation. The animals were then sacrificed, and the brains harvested for histological work-up. For the third group, five animals were monitored longitudinally to study the effects of anti-VEGF treatment on tumors during growth. Baseline MRI and MRE images were acquired 8 days after tumor implantation. A first dose of 10 mg B20 anti-VEGF antibody i.p. was administered in all five animals immediately after baseline scanning on Day 8 post tumor implantation. A second imaging time point was scheduled 2 days later after which all animals received a second injection of 10 mg B20 anti-VEGF antibody i.p. immediately after scanning. Two days later, imaging was performed a third time and animals were sacrificed for histology.

### MRI and MRE imaging

Imaging was performed on a 7 T Bruker small animal MRI scanner (BioSpec, Ettlingen, Germany; gradient strength 660 mT/m). Before imaging, 100 μl gadopentetate dimeglumine contrast agent (Magnevist, Bayer Health Care LLC, Whippany, NJ, USA) was administered intraperitoneally. Anesthesia was then induced with 2.5% isoflurane in 100% O_2_ and maintained with 1–1.5% isoflurane in 100% O_2_ delivered via a nose cone during the imaging procedure. Respiration rate was constantly monitored (SA Instruments, Stony Brook, NY, USA) and body temperature was sustained using a heated water mattress. The animal was placed on a custom-built bed with the head fixed in a cage assembly as described previously [14]. The head cage is coupled to a rod, which is connected to an external transducer outside the MRI scanner. For MRE, the transducer transmits mechanical vibrations at a frequency of 1 kHz through the rod, leading to a rocking motion of the head cage. This causes the production of mechanical shear waves within the animal’s brain. The propagation of these waves is measured with a dedicated MRI sequence and the biomechanical properties are reconstructed from the displacement field.

First, standard calibration, pilot scans and shimming were performed. Then, a coronal T1w sequence was acquired (FLASH; TR/TE 250/5.4 ms; FOV 19.2 mm; matrix 192 × 192; 6 averages; 9 slices; 0.3 mm slice thickness; acquisition time 3 min 36 s) followed by a coronal T2w sequence (RARE; TR/TE 5000/56 ms; FOV 19.2 mm; matrix 192 × 192; 6 averages; 9 slices: 0.3 mm slice thickness; acquisition time 12 min). T1w images were acquired approximately 30 min after injection of contrast agent. Finally, MRE was performed using a customized multi-slice, single spin echo sequence [23] (TR/TE 900/29 ms; FOV 19.2 mm; matrix 64 × 64; 1 average; 8 wave phases; 9 slices; isotropic resolution 0.3 mm; acquisition time 23 min; vibration frequency 1 kHz). The slices of all sequences were positioned in the tumor bearing region and covered identical volumes.

### Image analysis and statistics

MRE data were reconstructed according to published algorithms [7, 24] and analyzed using dedicated in-house software (ROOT environment, CERN; Meyrin, Switzerland). MRE maps of the absolute value of the complex valued shear modulus |*G*^∗^| and of the normalized phase angle Y were used for all analyses. Here *G*^∗^ = *G*_*d*_ + *iG*_*l*_ and $Y=\frac{2}{\pi}\mathit{\arctan}\left(\frac{G_l}{G_d}\right)$, where *G*_*d*_ is the stiffness (or elasticity) and *G*_*l*_ is the viscosity (or loss modulus). As |*G*^∗^| comprises measures of both elasticity and viscosity, we refer to $\mid {G}^{\ast}\mid =\sqrt{G_d^2+{G}_l^2}$ as viscoelasticity. The phase angle Y provides a measure of the relative contributions of elasticity and viscosity in the shear modulus. At the extremes, the tissue is pure elastic (*Y* = 0) or pure viscous (*Y* = 1). Contrast-enhanced T1w and T2w images were displayed in the same software and used as anatomical references for MRE-maps. Regions of interest (ROI) covering the tumor were defined on the T1w images following the outer borders of contrast enhancement and then copied to the corresponding MRE-maps. Additional mirror ROIs were placed in the normal appearing brain tissue (NABT) of the contralateral hemisphere and in the tissue directly surrounding the tumor (“periphery”). The NABT-ROI had a similar size as the tumor ROI and was positioned in the respective contralateral location. The “peripheral” ROI covered an approximately 600 μm thick rim of tissue surrounding the tumor. All ROIs excluded the ventricles, if possible. Mean and standard deviation of viscoelasticity and the phase angle were calculated for all ROIs collectively.

Additionally, T1w and T2w images were analyzed using open source 3D Slicer software (version 4.6, www.slicer.org) [25]. Tumors were manually segmented by a neuroradiologist with experience in small animal MRI using the contrast-enhanced T1w images. Tumor volume was derived from this segmentation using Slicer’s label statistics module. T2w images were used for an observational analysis of edema, hemorrhage, mass effect and occlusive hydrocephalus.

### Histology

The histopathological work-up included animals from experimental groups 2 and 3. Mice were sacrificed by CO_2_ asphyxation and intracardially perfused with 30 ml saline followed by 10% neutral buffered paraformaldehyde (PFA; cat#HT501128-4 L, Sigma-Aldrich, St Louis, MO, USA). Brains were harvested, post-fixed in 10% neutral buffered PFA and then transferred to 30% sucrose. Frozen brains were cryo-sectioned into 30 μm coronal sections. These were collected into sets with 300 μm spacing allowing for whole tumor volume reconstruction. The sections were labeled as follows: blue channel – DNA stained with DAPI, green channel – endogenous copGFP present in live GBM cells, red channel – myelin (Fluoro-myelin stain, cat# F34652, Fisher Scientific, Pittsburgh, PA, USA) or CD31 with secondary antibody Alexa594 (cat#550274, BD Pharmingen, San Diego, CA, USA; 712–586-150 Jackson ImmunoResearch, West Grove, PA, USA) and CD31 (cat# MCA2388GA, Bio-Rad, Hercules, MA, USA) for blood vessel staining.

Whole slide images were captured with a motorized Nikon Eclipse Ti fluorescence microscope (Nikon, Melville, NY, USA) and edited in Nikon’s NIS software and open source ImageJ (NIH ImageJ, https://imagej.net). Histological images in representative locations for each MRE and MRI image were identified. Images were analyzed qualitatively to determine the numbers of viable tumor cells and blood vessels. Additionally, we quantified the amount of myelin signal associated with neuronal tracks within the tumor and normalized the amount of myelin signal to tumor size. For this, 5 sections from each animal in experimental group 2 were used. An automated quantification of area for each channel (red – myelin, green – copGFP, blue – DNA) and a colocalization of myelin signal and copGFP were performed in ImageJ. Histological data were then compared to imaging parameters.

### Statistical evaluation

Statistical analyses were performed with GraphPad Prism (version 7 for Windows, GraphPad Software, La Jolla, CA, USA). Survival analysis was performed according to the Kaplan-Meier product limit method. Depending on the experiment, repeated measures one- or two-way ANOVA with Bonferroni’s test for multiple comparisons were conducted for MRE-parameters and tumor volume. The Bonferroni’s test compared the mean values for MRE-parameters and tumor volume between the time points for treated animals and untreated controls. Adjusted *p*-values derived from this test for multiple comparisons are reported along with the *p*-values from one- and two-way ANOVA. For the comparison of amount of myelin signal within the tumors of treated and untreated animals a Mann-Whitney test was used.

## Results

### Anti-VEGF treatment prolongs animal survival and slows tumor growth

To establish whether MRE could be applied to monitor the effects of anti-angiogenic therapy in GBM, we first established an animal model with a robust response to anti-VEGF therapy. We treated the G9 intracranial xenograft mouse GBM model with B20 anti-VEGF antibody starting on day 4 post-tumor implantation and compared outcomes to untreated controls (Group 1, Fig. 1a). Treated animals survived significantly longer than untreated controls (median survival with and without treatment 24 and 10 days respectively; *p* = 0.0011; Fig. 1b).

**Fig. 2:**
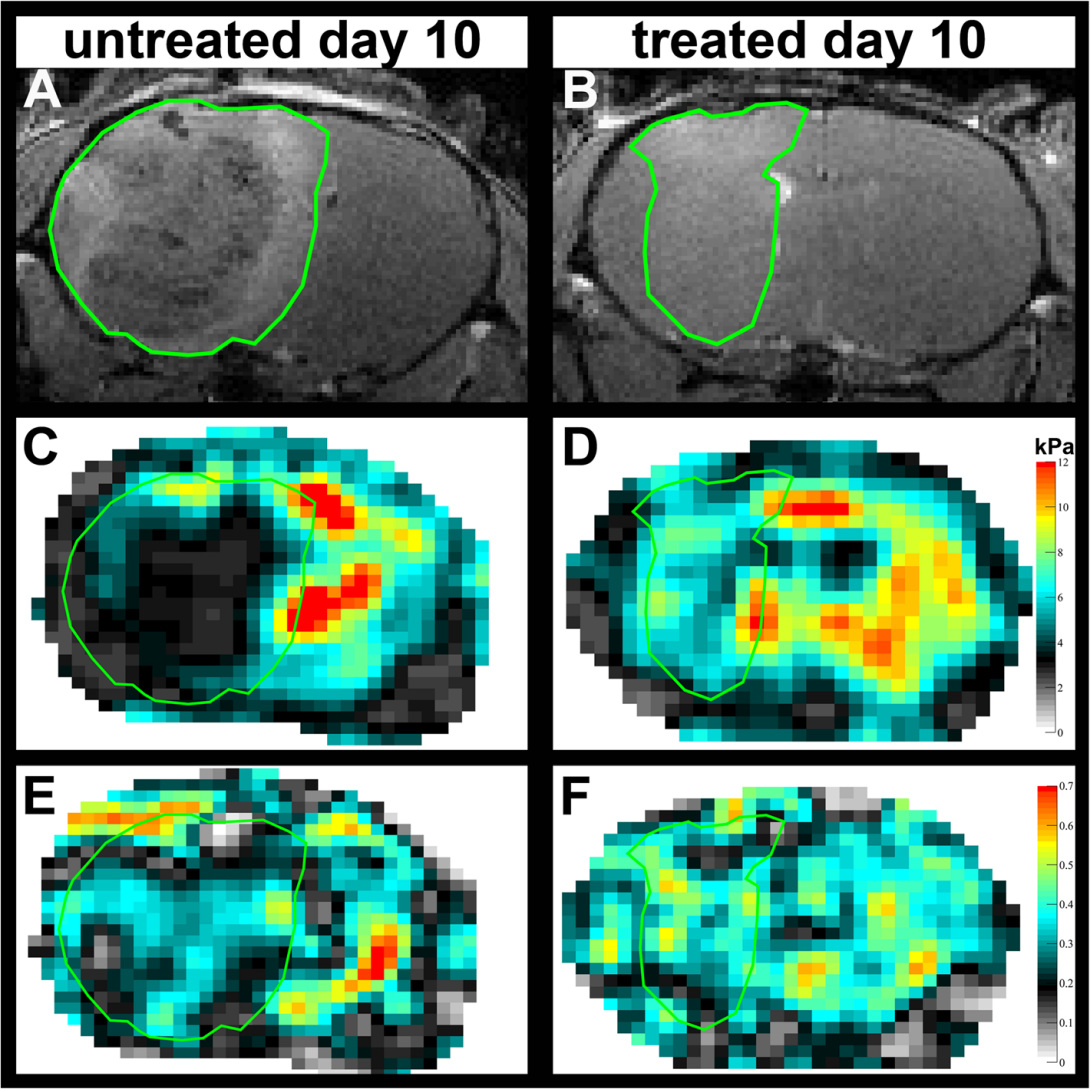
Contrast-enhanced T1w images (**a**, **b**) and the corresponding MRE-maps of viscoelasticity |G*| (**c**, **d**) and the phase angle Y (**e**, **f**) of an untreated control (A, C, E) and an animal treated with B20 anti-VEGF-antibody (B, D, F) 10 days after tumor implantation are shown. Both animals are from experimental group 2. The tumor is encircled in green. One can easily see that the untreated GBM (A) is larger than the treated one (B). Moreover, tumors in untreated animals were significantly softer (C) and had lower phase angles (E) compared to tumors in animals treated with B20 anti-VEGF-antibody (D, F)

**Fig. 3:**
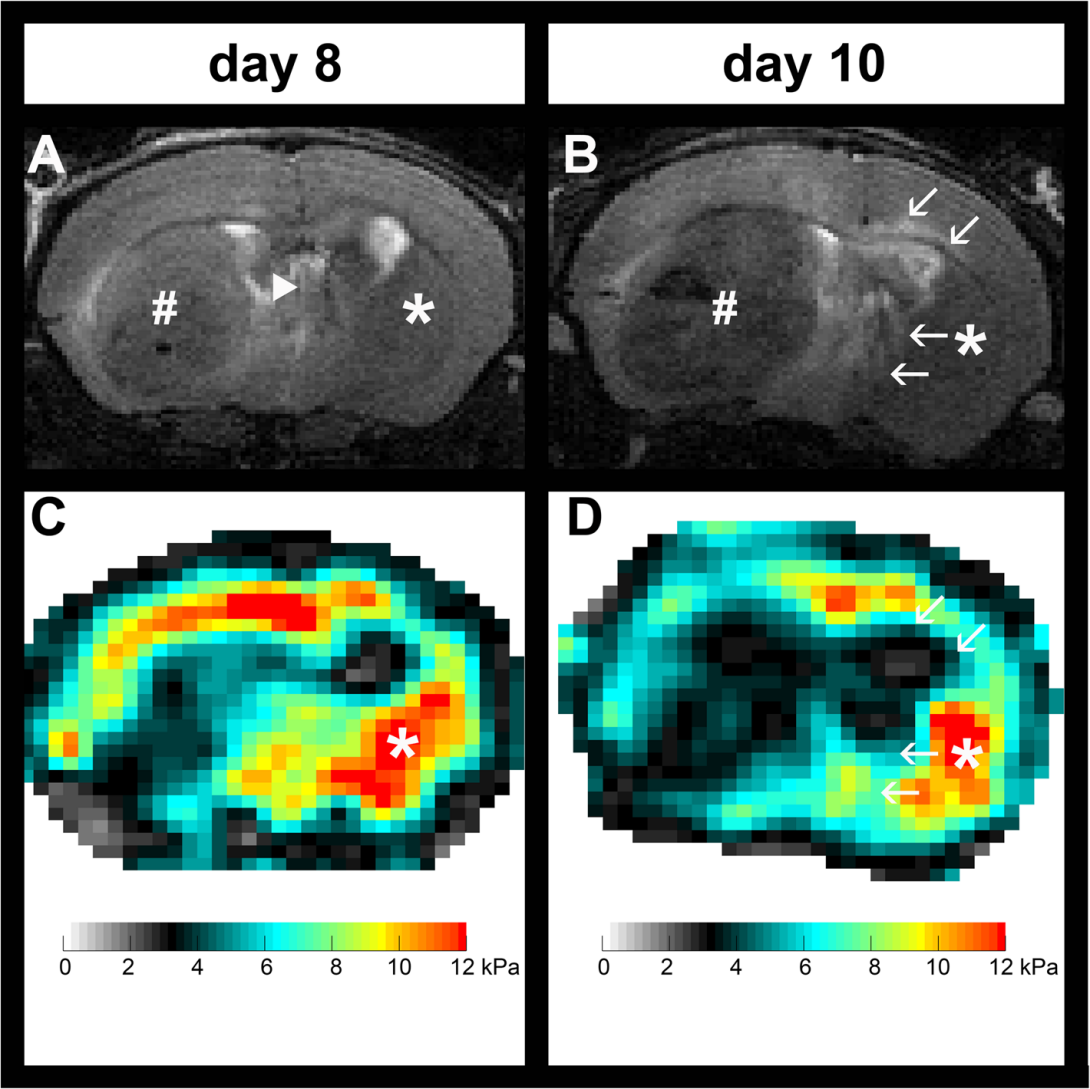
T2w images of an untreated animal from experimental group 2 at day 8 after tumor implantation. **a** Significant mass effect of the tumor (indicated by the #), which caused a midline shift (arrowhead in A; * indicates contralateral NABT). A T2w image of the same animal acquired 2 days later (**b**) revealed that the mass effect additionally led to an occlusive hydrocephalus with transependymal edema (arrows in B). Corresponding MRE-maps of viscoelasticity |G*| revealed a stiffening of the NABT on day 8 (**c**, NABT indicated by *), which regressed two days later (**d**, NABT indicated by *). The arrows in D indicate markedly soft areas, which correspond to the edema surrounding the enlarged lateral ventricle visible on the T2w image (B). The transient stiffening could be potentially related to an increased pressure due to the significant mass effect. The following softening could have been caused by an increased extracellular water content due to edema. NABT, normal appearing brain tissue

Further detailed studies were then performed using longitudinal imaging during anti-VEGF therapy. MRI revealed clear morphological differences between tumors in untreated controls and in animals treated with B20 anti-VEGF-antibody. The conventional T1w and T2w images show that tumors in treated animals were not only smaller but also appeared more homogeneous than in untreated controls (Fig. 2). Untreated tumors frequently exhibited intratumoral hemorrhage, which was present in all animals 10 days after tumor implantation. Moreover, untreated tumors caused a significant mass effect with midline shift, which was already visible at day 8 (Fig. 3). At day 10, additional transependymal edema as a consequence of occlusive hydrocephalus was present in untreated animals (Fig. 3). Tumor volumes of untreated controls and animals treated with anti-VEGF antibody were compared longitudinally (Group 2, Fig. 1a). Volumetric analysis using contrast-enhanced T1w images revealed significant differences between untreated and treated groups (*p* = 0.0001; Table 1; Fig. 2 a, b).

**Table 1:** Group 2 results. Two-way ANOVA followed by Bonferroni’s test for multiple comparisons comparing tumor volume, viscoelasticity |G*| and phase angle Y of tumors, NABT and the peripheral rim surrounding the tumor (“periphery”) between animals treated with B20 anti-VEGF antibody and untreated controls. NABT, normal appearing brain tissue; SD, standard deviation

Results two-way ANOVA						Results Bonferroni’s post-test			
	sum of squares	degree of freedom	mean squares	F	*p*-value		mean 1 (SD)	mean 2 (SD)	adjusted *p*-value
**tumor volume**									
interaction	1883	2	941.4	64.79	< 0.0001	untreated	*in mm*^*3*^	*in mm*^*3*^	
time	3569	2	1785	122.8	< 0.0001	d6 vs. d8	25.6 (8.1)	46.2 (10.2)	< 0.0001
treatment	7614	1	7614	50.17	0.0001	d6 vs. d10	25.6 (8.1)	71.6 (11.4)	< 0.0001
						d8 vs. d10	46.2 (10.2)	71.6 (11.4)	< 0.0001
						treated			
						d6 vs. d8	12.2 (4.4)	16.0 (4.0)	0.42
						d6 vs. d10	12.2 (4.4)	19.6 (5.0)	0.02
						d8 vs. d10	16.0 (4.0)	19.6 (5.0)	0.47
viscoelasticity |G*| tumor									
interaction	1.46	2	0.73	2.50	0.11	untreated	*in kPa*	*in kPa*	
time	7.49	2	3.74	12.87	0.0014	d6 vs. d8	6.8 (0.7)	6.0 (0.6)	0.6
treatment	6.34	1	6.34	8.12	0.0215	d6 vs. d10	6.8 (0.7)	5.1 (0.7)	0.0005
						d8 vs. d10	6.0 (0.6)	5.1 (0.7)	0.04
						treated			
						d6 vs. d8	7.1 (0.7)	7.2 (0.3)	> 0.9
						d6 vs. d10	7.1 (0.7)	6.4 (0.9)	0.2
						d8 vs. d10	7.2 (0.3)	6.4 (0.9)	0.09
**phase angle Y tumor**									
interaction	0.01	2	0.01	3.16	0.07	untreated	*normalized*	*normalized*	
time	0.01	2	0.01	7.18	0.0078	d6 vs. d8	0.33 (0.02)	0.30 (0.01)	0.04
treatment	0.01	1	0.01	12.84	0.0071	d6 vs. d10	0.33 (0.02)	0.29 (0.03)	0.02
						d8 vs. d10	0.30 (0.01)	0.29 (0.03)	> 0.9
						treated			
						d6 vs. d8	0.35 (0.02)	0.36 (0.03)	> 0.9
						d6 vs. d10	0.35 (0.02)	0.33 (0.03)	0.15
						d8 vs. d10	0.36 (0.03)	0.33 (0.03)	0.04
viscoelasticity |G*| NABT									
interaction	1.73	2	0.87	8.62	< 0.01	untreated	*in kPa*	*in kPa*	
time	2.43	2	1.21	12.06	< 0.01	d6 vs. d8	7.7 (0.22)	8.5 (0.37)	0.0045
treatment	1.33	1	1.33	3.36	0.99	d6 vs. d10	7.7 (0.22)	7.2 (0.34)	0.08
						d8 vs. d10	8.5 (0.37)	7.2 (0.34)	< 0.0001
						treated			
						d6 vs. d8	7.7 (0.56)	7.9 (0.53)	0.8
						d6 vs. d10	7.7 (0.56)	7.8 (0.56)	> 0.9
						d8 vs. d10	7.9 (0.53)	7.8 (0.56)	> 0.9
**phase angle Y NABT**									
interaction	0.01	2	0.01	3.07	0.07	untreated	*normalized*	*normalized*	
time	0.01	2	0.01	0.27	0.76	d6 vs. d8	0.40 (0.02)	0.37 (0.04)	0.16
treatment	0.01	1	0.01	0.56	0.47	d6 vs. d10	0.40 (0.02)	0.37 (0.04)	0.42
						d8 vs. d10	0.37 (0.04)	0.37 (0.04)	> 0.9
						treated			
						d6 vs. d8	0.37 (0.02)	0.39 (0.01)	0.88
						d6 vs. d10	0.37 (0.02)	0.39 (0.02)	0.64
						d8 vs. d10	0.39 (0.01)	0.39 (0.02)	> 0.9
viscoelasticity |G*| periphery									
interaction	0.48	2	0.24	0.55	0.59		*untreated (in kPa)*	*treated (in kPa)*	
time	3.6	2	1.8	4.16	0.04	untreated vs. treated d6	7.1 (0.54)	6.99 (0.31)	> 0.9
treatment	0.17	1	0.17	0.22	0.65	untreated vs. treated d8	7.3 (0.89)	7.4 (0.59)	> 0.9
						untreated vs. treated d10	6.3 (1.12)	6.7 (0.69)	0.9
**phase angle Y periphery**									
interaction	0.01	2	0.01	1.31	0.30		*untreated*	*treated*	
time	0.01	2	0.01	1.69	0.22	untreated vs. treated d6	0.33 (0.01)	0.34 (0.03)	> 0.9
treatment	0.01	1	0.01	0.90	0.08	untreated vs. treated d8	0.31 (0.02)	0.34 (0.04)	0.47
						untreated vs. treated d10	0.32 (0.02)	0.31 (0.01)	> 0.9

**Fig. 4:**
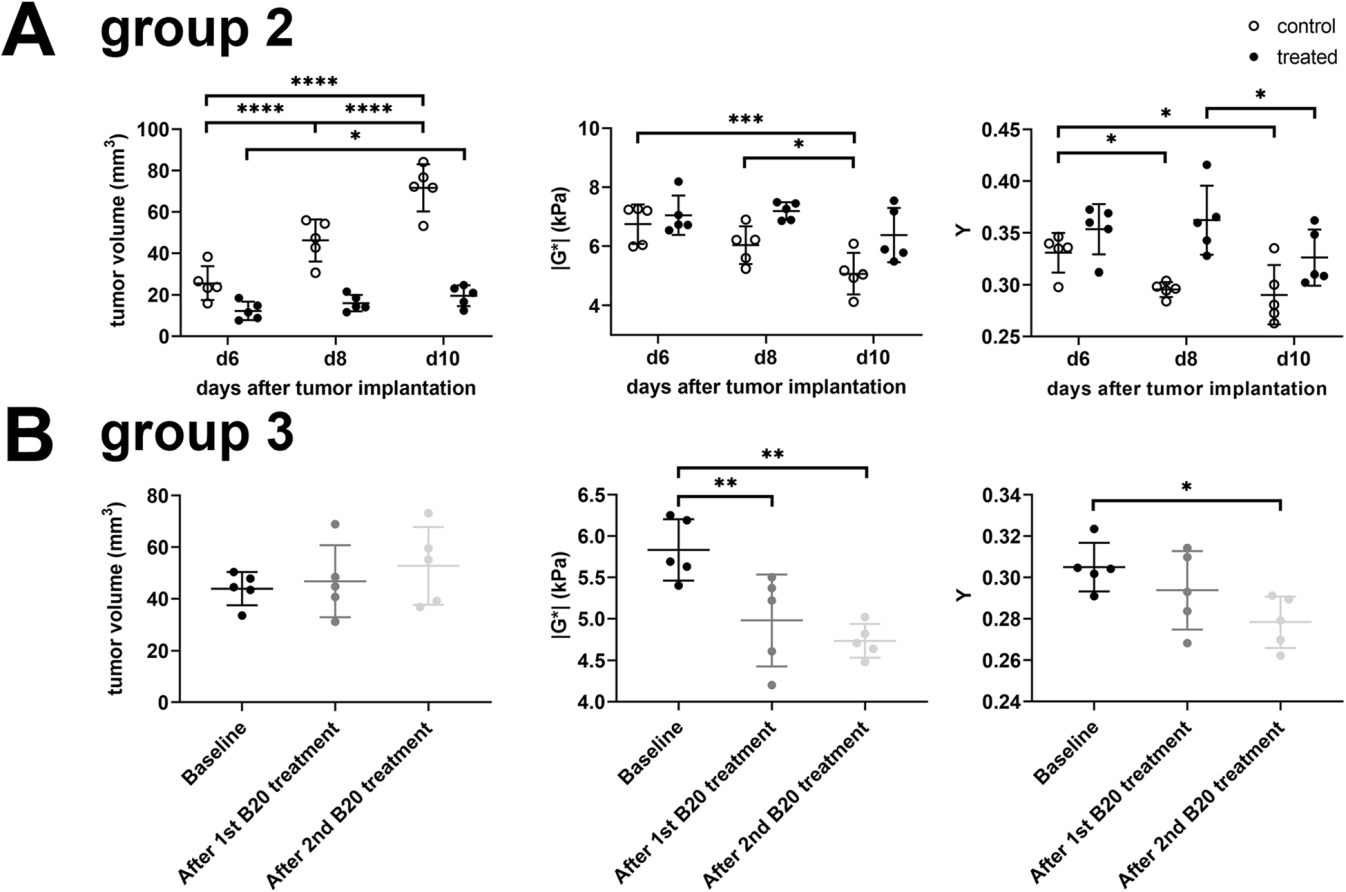
Graphs showing the temporal evolution of tumor volume, viscoelasticity |G*| and phase angle Y are shown for experimental group 2 (**a**) and group 3 **(b**). Volumetric analysis of group 2 data showed that tumor volume largely increased in untreated animals over time (A, controls depicted as white dots). In contrast, tumor volume remained almost stable under treatment (A, treated animals depicted as black dots). Viscoelasticity |G*| of the tumor progressively decreased in untreated animals (A, white dots), while it remained almost stable in animals treated with the B20 anti-VEGF-antibody (A, black dots). Similarly, the phase angle Y progressively decreased in untreated tumors (A, white dots). Of note, a decrease of Y is observable in treated animals 10 days after tumor implantation (A, black dots). Lines represent mean and standard deviation. Asterisks indicate the level of significance derived from a two-way ANOVA followed by Bonferroni’s test for multiple comparisons. The effects of anti-angiogenic treatment on established tumors were investigated in a separate experiment (group 3, B). Tumor volume, viscoelasticity |G*| and phase angle Y were compared at baseline (black dots in B), and after 1st (dark grey dots in B) and after 2nd administration (light grey dots in B) of B20 anti-VEGF antibody corresponding to days 8, 10 and 12 after tumor implantation, respectively. Tumor volume did not significantly increase over time. Both viscoelasticity and phase angle decreased over time. Lines represent mean and standard deviation. Asterisks indicate the level of significance derived from a one-way ANOVA followed by Bonferroni’s test for multiple comparisons

While tumor volume largely increased in untreated animals over time (mean tumor volumes on days 6, 8 and 10: 25.6 mm^3^, 46.2 mm^3^ and 71.6 mm^3^; *p* <  0.0001 between each time point; Table 1; Fig. 4a), tumor volume initially remained stable during anti-VEGF treatment and only significantly increased 10 days after tumor implantation (mean tumor volumes on days 6, 8 and 10: 12.1 mm^3^, 16.0 mm^3^ and 19.6 mm^3^; *p* = 0.02 when comparing the first to the last time point, with no significant differences for all other comparisons; Table 1; Fig. 4a).

To understand the potential utility of MRE in monitoring the effects of anti-VEGF treatment in our GBM model, we performed an in vivo study in which treatment was performed on well-established tumors (day 8 post intracranial tumor cell injection; Group 3, Fig. 1a). This allowed us to readily obtain baseline pre-treatment images from the tumors, which were well-established at day 8. Similar to the above studies, anti-VEGF treatment slowed tumor growth, with a non-significant increase in tumor volume as determined on contrast-enhanced T1w MR images comparing baseline volumes to those after the first and second treatment (mean tumor volumes 43.9 mm^3^, 46.8 mm^3^ and 52.7 mm^3^; adjusted *p*-values from Bonferroni’s test for multiple comparisons = 0.9 and 0.15 respectively. Table 2; Fig. 4b). Thus, anti-VEGF treatment leads to improved animal survival in the G9 xenograft GBM model, with a decrease in tumor volume observed when treatment was started either at day 4 (Group 2) or day 8 (Group 3) post-tumor implantation.

**Table 2:** Group 3 results. Comparison between tumor volume, viscoelasticity |G*| and phase angle Y of tumors, NABT and the peripheral rim surrounding the tumor (“periphery”) at baseline and after 1st and 2nd treatment with B20 anti-VEGF-antibody. The p-values are derived from a one-way ANOVA followed by Bonferroni’s test for multiple comparisons. BL, baseline NABT, normal appearing brain tissue; SD, standard deviation

Results one-way ANOVA						Results Bonferroni’s post-test			
	sum of squares	degree of freedom	mean squares	F	*p*-value		mean 1 (SD)	mean 2 (SD)	adjusted *p*-value
**tumor volume**							*in mm*^*3*^	*in mm*^*3*^	
treatment	202.2	2	101.1	2.74	0.1241	BL vs. after 1st B20 treatment	43.9 (6.4)	46.8 (14.0)	> 0.9
individual	1559	4	389.7	10.56	0.0028	BL vs. after 2nd B20 treatment	43.9 (6.4)	52.7 (15.1)	0.15
						After 1st vs. after 2nd B20 treatment	46.8 (14.0)	52.7 (15.1)	0.48
viscoelasticity |G*| tumor							*in kPa*	*in kPa*	
treatment	3.32	2	1.67	19.47	0.0008	BL vs. after 1st B20 treatment	5.8 (0.4)	5.0 (0.6)	0.0052
individual	1.26	4	0.31	3.68	0.0551	BL vs. after 2nd B20 treatment	5.8 (0.4)	4.7 (0.2)	0.0010
						After 1st vs. after 2nd B20 treatment	5.0 (0.6)	4.7 (0.2)	0.66
**phase angle Y tumor**							*normalized*	*normalized*	
treatment	0.002	2	0.001	6.03	0.0467	BL vs. after 1st B20 treatment	0.31 (0.01)	0.29 (0.02)	0.45
individual	0.001	4	0.001	2.38	0.14	BL vs. after 2nd B20 treatment	0.31 (0.01)	0.28 (0.01)	0.04
						After 1st vs. after 2nd B20 treatment	0.29 (0.02)	0.28 (0.01)	0.58
viscoelasticity |G*| NABT							*in kPa*	*in kPa*	
treatment	3.19	2	1.59	3.88	0.07	BL vs. after 1st B20 treatment	8.1 (0.6)	7.1 (0.6)	0.09
individual	0.82	4	0.20	0.50	0.74	BL vs. after 2nd B20 treatment	8.1 (0.6)	7.3 (0.6)	0.21
						After 1st vs. after 2nd B20 treatment	7.1 (0.6)	7.3 (0.6)	> 0.9
**phase angle Y NABT**							*normalized*	*normalized*	
treatment	0.003	2	0.002	7.28	0.02	BL vs. after 1st B20 treatment	0.35 (0.02)	0.39 (0.01)	0.02
individual	0.003	4	0.001	3.56	0.06	BL vs. after 2nd B20 treatment	0.35 (0.02)	0.37 (0.03)	0.19
						After 1st vs. after 2nd B20 treatment	0.39 (0.01)	0.37 (0.03)	0.41
viscoelasticity |G*| periphery							*in kPa*	*in kPa*	
treatment	1.00	2	0.51	3.06	0.10	BL vs. after 1st B20 treatment	6.6 (0.4)	6.0 (0.4)	0.13
individual	1.88	4	0.47	2.87	0.10	BL vs. after 2nd B20 treatment	6.6 (0.4)	6.2 (0.7)	0.36
						After 1st vs. after 2nd B20 treatment	6.0 (0.4)	6.2 (0.7)	> 0.9
**phase angle Y NABT**							*normalized*	*normalized*	
treatment	0.001	2	< 0.001	0.30	0.75	BL vs. after 1st B20 treatment	0.31 (0.01)	0.30 (0.02)	> 0.9
individual	0.002	4	< 0.001	1.52	0.28	BL vs. after 2nd B20 treatment	0.31 (0.01)	0.31 (0.02)	> 0.9
						After 1st vs. after 2nd B20 treatment	0.30 (0.02)	0.31 (0.02)	> 0.9

### MRE-defined biomechanical tumor properties are different in tumors treated with anti-angiogenic therapy and change over time

In parallel to the MRI measurements of tumor volume described above, MRE was used to interrogate the biomechanical tumor properties in terms of viscoelasticity |*G*^∗^| and the phase angle Y. These parameters were compared between untreated and treated animals.

This comparison revealed that anti-VEGF treatment starting at day 4 post-tumor implantation affects tumor viscoelasticity as tumors in untreated animals were significantly softer and had lower values of the phase angle Y. Lower phase angle indicates that the tumor behaves more like an elastic material, compared to tumors in animals treated with B20 anti-VEGF-antibody (*p* = 0.02 for |*G*^∗^| and *p* = 0.0071 for Y; Table 1; Fig. 2d, f and Fig. 4a). Moreover, the viscoelasticity of the tumor progressively decreased in untreated control animals (mean |*G*^∗^| of tumor on days 6, 8 and 10: 6.8 kPa, 6.0 kPa and 5.1 kPa; *p* = 0.0005 when comparing day 6 with day 10 and *p* = 0.04 when comparing day 8 with day 10; Table 1; Fig. 4a). In contrast, viscoelasticity showed a trend to decrease in animals treated with the B20 anti-VEGF-antibody but without reaching significance (mean |*G*^∗^| of tumor on days 6, 8 and 10: 7.1 kPa, 7.2 kPa and 6.4 kPa; *p* >  0.05 for each comparison; Table 1; Fig. 4a). In line with viscoelasticity, the phase angle Y progressively decreased in untreated tumors (mean Y of tumor on days 6, 8 and 10: 0.33, 0.30 and 0.29; adjusted *p*-value from Bonferroni’s test for multiple comparisons *p* = 0.04 when comparing day 6 with day 8 and *p* = 0.02 when comparing day 6 with day 10; Table 1; Fig. 2c, e and Fig. 4a). The phase angle decreased in treated animals 10 days after tumor implantation (mean Y of tumor on days 6, 8 and 10: 0.35, 0.36 and 0.33; *p* = 0.04 when comparing day 8 with day 10, *p* >  0.05 for all other comparisons; Table 1; Fig. 4a).

In experimental Group 3, following treatment from the day 8 baseline in established tumors, viscoelasticity and the phase angle also decreased over time after treatment (*p* <  0.0008 for |*G*^∗^| and *p* = 0.0467 for Y). Specifically, significant differences in the viscoelasticity of tumors could be observed between baseline and after the first and second treatment (mean |*G*^∗^| of tumor: 5.8 kPa, 5.0 kPa and 4.7 kPa; *p* = 0.0052 and *p* = 0.001 when comparing the measurements after the first and second treatment, respectively compared with baseline; Table 2; Fig. 4b). Values of the phase angle only differed from baseline after the second treatment (mean Y of tumor at baseline and after first and second treatment: 0.31. 0.29 and 0.28; *p* = 0.04 when comparing baseline with after second treatment; Table 2; Fig. 4b). In summary, viscoelasticity |G*| and the phase angle Y progressively decreased in untreated controls. In animals treated with B20 anti-VEGF antibody, there was a trend of a decrease in viscoelasticity, while the phase angle Y significantly decreased at the last imaging time point. The comparison of baseline to tumors during the course of treatment revealed a similar evolution of the MRE-parameters to untreated controls. The magnitude of this change was lower than when treatment began at day 4, likely due to reduced effects of therapy when starting in established tumors at day 8.

### MRE does not capture treatment effects on tissue in the direct tumor vicinity, but depicts tumor-related changes in NABT

We investigated additionally whether the biomechanical properties of the tissue in the direct tumor vicinity and of NABT of the contralateral hemisphere were different when comparing untreated and treated tumors (Group 2) or baseline (prior to treatment) compared to subsequent treatment (Group 3). The viscoelasticity of the rim of surrounding tissue decreased slightly over time in both untreated and treated animals (*p* = 0.04; Table 1). However, no significant differences could be observed between the groups and when comparing baseline viscoelastic values to those after first or second treatment (*p* >  0.05 each; Tables 1 and 2). The phase angle did not differentiate either between the peripheral tumor rim of untreated and treated animals or between baseline and post-treatment values (*p* >  0.05 each; Tables 1 and 2).

Untreated animals presented with a transient stiffening of the NABT at day 8 (mean |*G*^∗^| in untreated animals at days 6, 8 and 10: 7.7 kPa, 8.5 kPa and 7.2 kPa; *p* = 0.0045 when comparing day 6 with day 8 and *p* <  0.0001 when comparing day 8 with day 10; Table 1; Fig. 3). Of note, T2w images of untreated animals at day 8 showed a significant mass effect of the tumors, which caused a midline shift (Fig. 3). At day 10, the mass effect additionally led to an occlusive hydrocephalus with transependymal edema (Fig. 3). This was not accompanied by significant changes in the phase angle (Y of NABT in untreated animals at days 6, 8 and 10: 0.40, 0.37 and 0.37; *p* >  0.05 for each comparison; Table 1). Treated animals did not show any alterations of the biomechanical NABT properties (Table 1). Additionally, the viscoelasticity of the NABT at baseline remained stable after both the first and second treatments (*p* >  0.05 each; Table 2). The phase angle Y increased in the NABT after the first treatment with B20 anti-VEGF antibody and then remained stable (Y of NABT at baseline and after first and second treatment with B20: 0.35, 0.39 and 0.37; *p* = 0.02 when comparing baseline to after first B20-treatment; Table 2).

### Anti-angiogenic treatment normalizes vasculature and partially preserves brain composition within the tumor

**Fig. 5:**
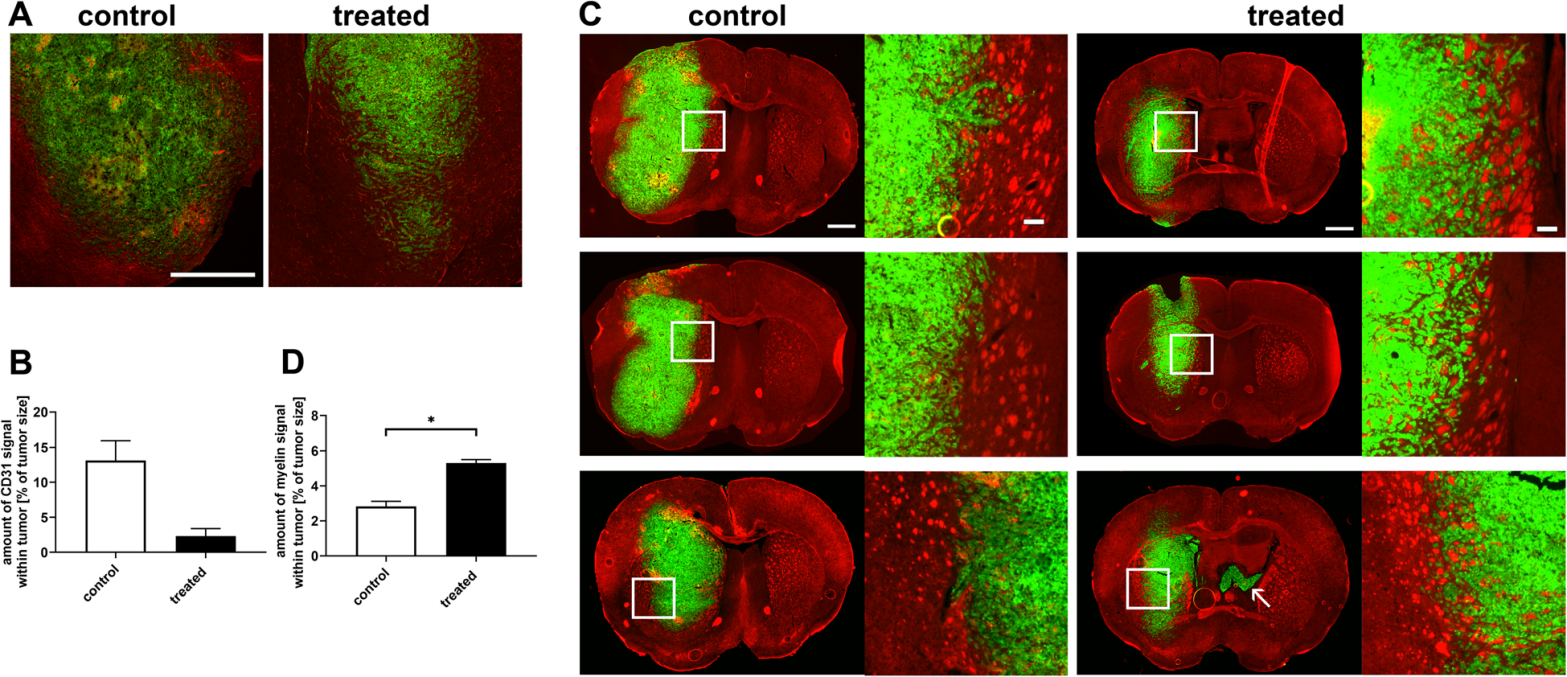
Histological studies of GBM after anti-angiogenic treatment. Immunofluorescence for GBM cells (green in A and C) and blood vessels (red in A) or myelin (red in C) exposed marked differences between untreated and treated tumors. In untreated controls, blood vessels (red) were abundant and larger than in the contralateral hemisphere, especially in the peripheral parts of the tumor (**a**). In contrast, blood vessels in animals treated with B20 anti-VEGF-antibody were accentuated in the tumor center, but otherwise not significantly different from NABT in the remaining tumor (A). Scale bar = 1 mm. The direct comparison of the amount of blood vessels in an untreated control and a treated animal showed that the overall amount of blood vessels was smaller after anti-angiogenic treatment (**b**). The graph depicts the percentage of blood vessel signal within the tumor of an untreated control (white box) and a treated animal (black box). Mean and SD are shown. Myelin staining showed marked differences between treated tumors and controls (**c**). Immunofluorescence for GBM cells (green) and myelin (red) exposed marked differences between untreated and treated tumors. Sections of three untreated controls and three animals treated with B20 anti-VEGF-antibody from experimental group 2 in similar brain regions are shown in total. The boxes (1.5 mm^2^) indicate the areas which are magnified in the right column in B and C. Treated tumors consisted of densely packed GBM cells (green), which were relatively homogeneous distributed over the entire tumor area and were spread around myelinated fiber bundles. The center of untreated tumors was more heterogeneous than the periphery and less myelin was identifiable between the tumor cells. The borders of untreated tumors were relatively well circumscribed, while clusters of GBM cells were observable around the margins of the main tumor mass Furthermore, tumor cell infiltration of the ventricles was observable in three treated animals (exemplary shown in C, left panel, bottom row and marked with an arrow). The amount of myelin within the tumors was quantified by automatically counting myelin bundles within the tumor and normalizing the amount of myelin signal to tumor size. The graph (**d**) depicts that in tumors of treated animals (black box) the myelin signal was higher than in untreated controls (white box). Mean and SEM are shown, the asterisk indicates the level of significance derived from a Mann-Whitney test

To investigate which molecular or structural features underlie the observed differences in MRE parameters, we compared the histological tumor features of untreated and treated animals. Conventional H&E staining revealed densely packed cells in the right striatum of all animals corresponding to the tumor (Fig. 5a). Control animals presented with larger tumors than treated ones, which were more expansive and led to a relevant displacement of surrounding tissues such as the ventricles.

Immunofluorescent staining for DNA, tumor cells, myelin and blood vessels revealed marked phenotypic differences between untreated and treated tumors. Staining of tumor associated vasculature using CD31 showed extensive abnormalities in control tumors. These areas were extensive within the core of the tumor, and represent areas where vascular leakage occurs with subsequent necrosis. As expected anti-VEGF treatment normalized tumor vasculature, with less blood vessels overall, and much less apparent abnormal vasculature (Fig. 5a). In addition to that, the overall amount of blood vessels within the tumor of an animal treated with anti-VEGF-antibody was less than in an untreated control animals (Fig. 5b).

Treated tumors consisted of densely packed GBM cells, which were relatively homogeneously distributed over the entire tumor area and were spread around myelinated fiber bundles. In contrast, the center of untreated tumors was more heterogeneous than the periphery and less myelin was identifiable within the tumors. The borders of untreated tumors were relatively well circumscribed (Fig. 5c) and could be easily distinguished from surrounding brain tissue. In treated animals however, clusters of GBM cells were observable around the margins of the main tumor mass (Fig. 5c). Furthermore, tumor cell infiltration of the ventricles was observed in three treated animals. This is in agreement with previous studies showing enhanced invasion in murine GBM models treated with anti-VEGF [18, 26].

In order to further quantify the more invasive growth pattern of GBM treated with B20 anti-VEGF antibody in contrast to tumors in untreated controls with a more destructive growth pattern and compression of the surrounding brain tissue, we used automated image analysis to quantitate the amount of myelin within the tumors as a measure of preserved brain structure. In treated animals, there were more myelinated structures identifiable within the tumors than in untreated controls (mean percentage of myelin in the whole tumor - defined as amount of myelin signal within the tumor normalized to tumor size - 5.3% in treated and 2.8% in untreated control animals; *p* = 0.03; Fig. 5d). Thus, the growth pattern of GBM in treated animals was more invasive than in untreated controls, with increased preservation of the normal brain composition. Malignant tumors have been shown to be softer than healthy brain parenchyma [9] and loss of myelin leads to a softening of brain tissue [27]. Based on this, the partial preservation of stiff myelin structures within GBM in treated animals leads to higher values of tumor viscoelasticity than in untreated controls, where the normal brain architecture is destroyed.

When examining the tumors of animals that received anti-angiogenic treatment in established tumors (Group 3), histological features similar to both treated and untreated tumors from Group 2 were identified. Tumors in these animals were large and had a heterogeneous center like in untreated controls, but showed satellite tumors in the periphery comparable to those observed in treated animals in Group 2. Thus, our data shows for the first time that MRE can detect changes in GBM tumor growth based on responses to anti-VEGF treatment. This data provides a foundation for further more detailed studies in additional preclinical models, to determine potential applicability in human patients.

## Discussion

Radiological monitoring of response to anti-angiogenic treatment of GBM is challenging and often hindered by phenomena such as pseudoresponse [28]. This can occur rapidly after initiation of anti-angiogenic therapy with the anti-VEGF monoclonal antibody bevacizumab and is due to a normalization of the vasculature [19, 29]. As radiological response assessment is based on the evaluation of contrast enhancing tumor components [4], follow-up scans are needed to differentiate pseudoresponse from true tumor response. Hence, new imaging biomarkers need to be established to aid reliable response assessment and to help tailor treatment strategies to the individual patient.

In our study, treatment with the B20 anti-VEGF antibody significantly prolonged survival of animals with GBM and slowed tumor growth. G9pCDH is a highly aggressive GBM cell line and the typical survival time of animals is only 12–14 days without treatment. Rubenstein and colleagues observed a similar effect of anti-angiogenic treatment in an orthotopic GBM model in athymic rats [26]. Keunen et al. investigated the response of GBM to bevacizumab in an intracranial rat xenograft model using comprehensive MRI and histopathology [30]. They observed that GBM in animals treated with bevacizumab were smaller, exhibited less contrast-enhancement and had significantly reduced tumor blood flow and volume [30]. In contrast, addition of bevacizumab to radiotherapy/temozolomide in human patients with GBM had no significant effect on overall survival [31]. However, bevacizumab does significantly affect time-to-progression and maintains baseline quality-of-life as well as performance status [31], and is widely used in GBM therapy, particularly as a second-line treatment and in recurrent GBM. Thus, in humans bevacizumab appears to have profound effects on tumor biology even though ultimately the tumor escapes. The model used in this study was chosen as a proof-of principle to understand whether biological effects of an anti-VEGF antibody (vascular normalization, increased tumor invasion) could be detected using a novel imaging modality (MRE). Previous unpublished experiments have shown a rapid growth in nu/nu mice with the presence of tumor core (displaying necrotic areas) and partially invasive edge of tumor mass. Additionally, G9pCDH showed response to anti-angiogenic treatment. As this is the first study to investigate the effects of any drug treatment on the biomechanical properties of GBM, we chose the G9 model intentionally to see if a tumor model known to be responsive to anti-angiogenic treatment shown in the survival experiment can be depicted in MRE. The G9 model is based on a patient-derived GBM neurosphere culture, and as such represents the state-of-the art in the field.

**Fig. 6:**
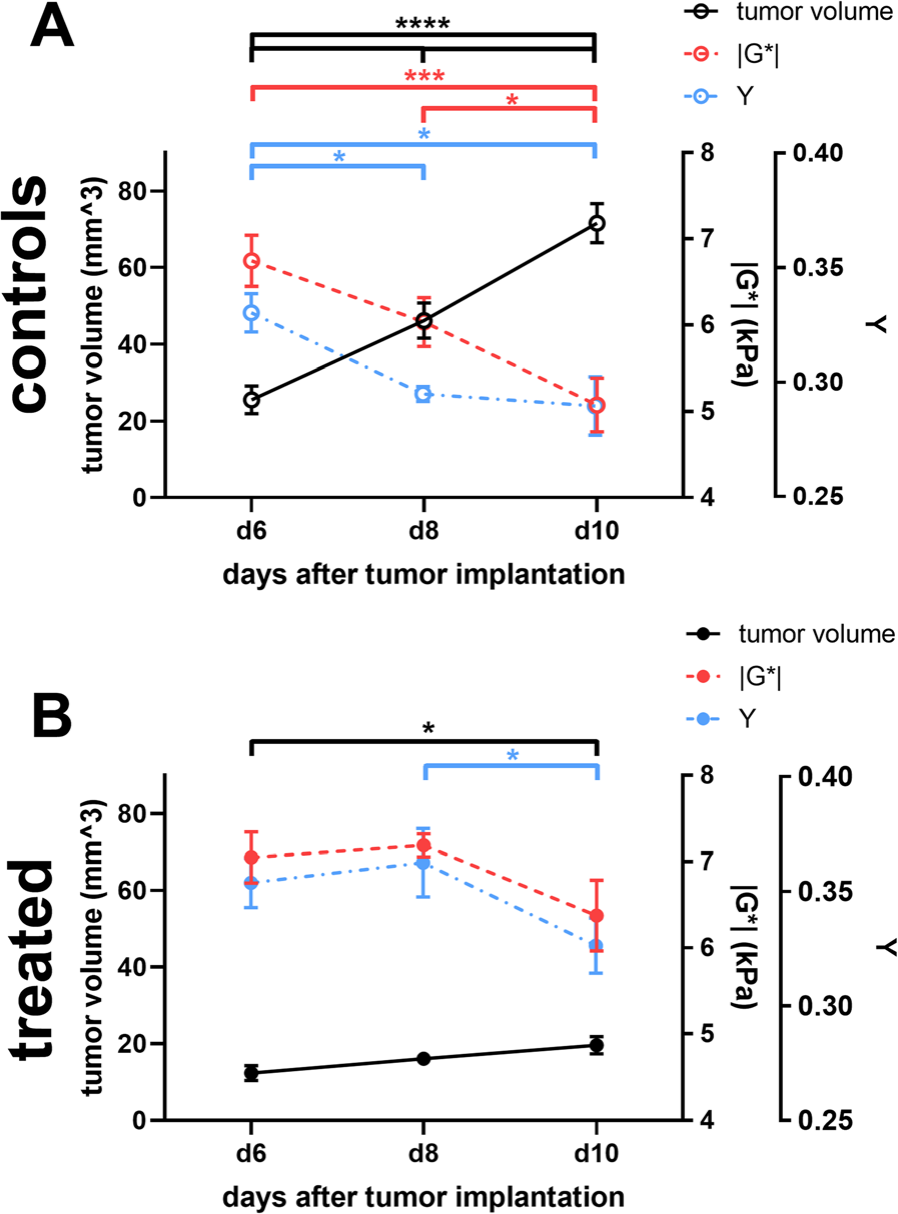
Graphs depicting the temporal evolution of tumor volume (black dots), MRE-parameters |G*| (red dots) and phase angle Y (blue dots) of the tumors are shown for untreated control animals (**a**) and animals treated with B20 anti-VEGF-antibody (**b**). In controls (A), tumor volume progressively increased, while |G*| and Y decreased. During anti-angiogenic treatment (B), tumor volume only increased at the last time point. |G*| trended to decrease. The phase angle Y significantly decreased 10 days after tumor implantation. Lines represent means and standard error of the mean. No error appears for tumor volume in treated animals at day 8 after tumor implantation, as the standard error is so small that the error bar would be shorter than the size of the symbol. Asterisks in A and B indicate the level of significance derived from a two-way ANOVA followed by Bonferroni’s test for multiple comparisons

Our results show that MRE is able to detect effects of anti-angiogenic treatment with the B20 anti-VEGF antibody on biomechanical tumor properties and to differentiate treated and untreated animals. GBM in untreated control animals progressively increased in size, while viscoelasticity |G*| as well as the phase angle Y decreased (Fig. 6). The progressive decrease of the biomechanical properties is likely caused by destruction of the normal brain composition leading to tissue softening and an overall heterogeneous tumor structure. Anti-angiogenic treatment with the B20 anti-VEGF antibody decelerated these processes leading to a more homogeneous histological tumor composition. The growth pattern was more invasive as has been previously shown [18, 26] and normal brain structures such as myelinated fiber bundles were preserved much better than in untreated controls. Malignant intracranial tumors have been described to be softer than healthy brain parenchyma in both animal models [15, 16] and human patients [10, 11, 13]. Moreover, an increasing softening was observed with tumor progression [14, 16]. In line with this, tumors in our study were softer than NABT. Importantly, the progressive decrease of viscoelasticity is decelerated by anti-angiogenic treatment. Nonetheless the viscoelasticity of treated tumors continued to decrease after initiation of treatment, paralleling the course observed in untreated animals, however to a lesser extent. The higher amount of myelin structures observed within treated tumors might explain the differences observed in viscoelasticity between animals during anti-angiogenic treatment and untreated controls. In treated tumors, the normal brain composition was better preserved than in untreated controls leading to higher viscoelasticity. Studies showing a decrease of viscoelasticity with demyelination in a mouse model [27] and in patients with multiple sclerosis [32–34] corroborate this hypothesis.

The phase angle Y decreased significantly in treated animals, while tumor volume slightly increased. In a study investigating the biomechanical properties of breast tumors, the phase angle Y discriminated well between benign and malignant breast lesions and seemed to indicate tumor aggressiveness [35]. The phase angle, which probes tissue integrity in a subtle manner [36], might be sensitive to progression of GBM escaping anti-angiogenic therapy. Results from a recent study suggest that the phase angle indicates tissue fluidity [37]. In that study, the fluid fraction of GBM, defined by the area in which fluid properties dominate normalized to total tumor area, was higher than that of healthy brain parenchyma and benign meningiomas, and the authors hypothesized that fluidity enables GBM to infiltrate in the periphery [37]. There is evidence in the literature that GBM adapts to inhibition of angiogenesis by increased infiltration and co-option of the host vasculature [18, 26]. We also observed satellite GBM cell clusters around the tumor margins in treated animals and the amount of myelin was higher in treated animals than in untreated controls. This may be due to increased tumor invasiveness during anti-angiogenic treatment, which is potentially reflected by a decrease in the phase angle Y.

We observed satellite GBM cell clusters around the tumor margins in treated animals, which were not as pronounced in untreated controls. Even though slight decreases of the viscoelasticity in the tissue in the immediate tumor vicinity were observable over time, MRE was not able to unequivocally depict this increased invasiveness. A possible explanation for this is that the current spatial resolution of MRE with 300 μm isotropic is not sufficient to capture tumor cell invasion in the mouse brain. Future studies using MRE techniques focused on increased spatial resolution [38–40] in animals or humans with GBM or animal studies using GBM cell lines with a more extensive tumor cell invasion are warranted to clarify whether MRE could depict tumor invasiveness.

A transient stiffening of NABT occurred in untreated animals at day 8, which was followed by a decrease in the viscoelasticity at day 10. This stiffening could be potentially related to an increased pressure due to the significant mass effect with midline shift as visible on T2w images. The following softening could have been caused by occlusive hydrocephalus with transependymal edema at day 10. Similar observations have been made in a study which investigated tissue stiffness during the development of obstructive hydrocephalus in rats [41]: rapid enlargement of ventricles led to tissue compression and was associated with stiffening, while increased extracellular water content due to edema led to softening of the brain parenchyma.

The experimental set-up used in this study including MRE in a standard MRI imaging protocol can easily be transferred to other tumor models and could be of use for the exploration of biomechanical properties of other tumor types and different pathological conditions of the brain as well. Here, we demonstrated MRE parameters that are sensitive to growth of GBM in mice during anti-angiogenic treatment with an anti-VEGF-antibody. Limitations of our study include the small sample size and the use of only one tumor cell line. Further studies using a range of cell lines (resistant and sensitive) are needed to understand in more detail how anti-angiogenic treatment can be assessed using MRE. Furthermore, the effects of a treatment regimen combining other therapeutic agents and/or radiotherapy with anti-angiogenic agents on the biomechanical tumor properties should be investigated, as these represent the standard of care in therapy of patient with GBM. Finally, the transferability of findings derived in an animal model to patients should be tested.

## Conclusions

In conclusion, we showed that MRE can capture the treatment effects of a responsive GBM cell line in mice after treatment with the B20 anti-VEGF antibody; the rate of decrease of viscoelasticity was reduced in treated animals, which might be explained by altered blood vessels and a higher degree of preserved myelin within treated tumors. Results from this preliminary pilot study hint at the potential of MRE to prove valuable for treatment monitoring of patients with GBM complementing existing MRI techniques.

## Data Availability

The datasets analyzed during the current study are available from the corresponding authors on reasonable request.

## Abbreviations

FLAIR: fluid-Attenuated inversion recovery
GBM: Glioblastoma
IACUC: Institutional animal care and use committee
i.p.: Intraperitoneal
MRE: Magnetic resonance elastography
NABT: Normal appearing brain tissue
PFA: Paraformaldehyde
RANO: Response assessment in neuro-oncology
ROI: Region of interest
T1w: T1-weighted
T2w: T2-weighted
VEGF (−A): Vascular endothelial growth factor (A)
